# Pet Ownership and Maintenance of Physical Function in Older Adults—Evidence From the Baltimore Longitudinal Study of Aging (BLSA)

**DOI:** 10.1093/geroni/igac080

**Published:** 2022-12-25

**Authors:** Erika Friedmann, Nancy R Gee, Eleanor M Simonsick, Erik Barr, Barbara Resnick, Emily Werthman, Ikmat Adesanya

**Affiliations:** University of Maryland School of Nursing, Baltimore, Maryland, USA; Department of Psychiatry, School of Medicine, Virginia Commonwealth University, Richmond, Virginia, USA; Intramural Research Program, National Institute on Aging, National Institutes of Health, Baltimore, Maryland, USA; University of Maryland School of Nursing, Baltimore, Maryland, USA; University of Maryland School of Nursing, Baltimore, Maryland, USA; University of Maryland School of Nursing, Baltimore, Maryland, USA; University of Maryland School of Nursing, Baltimore, Maryland, USA

**Keywords:** Functional status, Healthy aging, Human–animal interaction, Leisure time activity, Physical performance

## Abstract

**Background and Objectives:**

Pet ownership or human–animal interaction has been associated with better health outcomes in individuals with disease or disability. We hypothesized that pet ownership, as well as dog ownership and cat ownership separately, are associated with maintaining physical function, and leisure time physical activity and that among dog owners, dog walking is associated with maintaining these outcomes for generally healthy community-dwelling older adults participating in the Baltimore Longitudinal Study of Aging.

**Research Design and Methods:**

A total of 637 men (44.1%) and women aged 50–100 years (*M* = 68.3, standard deviation [*SD*] = 9.6) completed a comprehensive pet ownership questionnaire that ascertained pet ownership history 10–13 years and had serial assessments of physical function every 1–4 years prior. Linear or generalized linear mixed models with time varying pet ownership were used to examine change in physical function over a mean of 7.5 years (range 1–13, *SD* = 3.6) according to pet ownership.

**Results:**

Pet owners (*n* = 185) were significantly younger (*p* < .001) and had fewer comorbidities (*p* = .03) than nonowners; thus, age and comorbidities were included as covariates in the longitudinal analyses. Physical function and leisure time physical activity declined with aging across all outcomes (*p* < .001); the decline was slower among pet owners in overall physical performance (*p* < .001), rapid gait speed (*p* = .03), usual gait speed (*p* = .032), cardiorespiratory fitness (*p* < .001), and physical well-being (*p* = .002) controlling for age and comorbidities. Changes in leisure time physical activities with aging did not differ between pet owners and nonowners. Dog walking was not independently related to the maintenance of physical function or leisure time physical activity with aging.

**Discussion and Implications:**

This study provides the first longitudinal evidence that pet ownership is associated with maintained physical function among community-dwelling generally healthy older adults.

**Translational Significance:** Prior research indicates that human–animal interaction promotes physical function of older adults residing in care communities. There is a dearth of data relating pet ownership to longitudinal changes in physical function for generally healthy older adults residing in community settings. Older adult pet owners experienced less decline in physical function as they aged, after considering both their preexisting health and age. Pet ownership was not related to changes in leisure time physical activity. Policy makers can use these outcomes to consider possible contributions of pet ownership in the design and assessment of strategies for maintaining physical function in this population.

Physical function typically decreases as adults age, with greater decreases occurring in the later years of life (Metti et al., 2018). Declines in function and increases in disease and disability frequently lead to poorer quality of life during the final years. As the proportion of the population in the older age groups increases over the next several decades, addressing older adults’ health and care needs will become a more pressing problem (Vespa, 2018). Promoting healthy aging is intended to reduce the current 5–7 year gap between high life quality and total life expectancy and enable people to live with the best function possible for as long as possible (Beltrán-Sánchez et al., 2015). Healthy aging depends on using many strategies to help minimize the declines in physical and cognitive function, in the development and progression of disease and disability, and in active engagement with life.

Human–animal interaction in several forms is suggested to promote physical, psychological, and social health in an aging population (Friedmann, 2019; Friedmann & Gee, 2018; Gee, 2021; Gee & Galik, 2019; Gee & Mueller, 2019; Gee et al., 2021). Pet ownership is common in community-dwelling older adults with estimates ranging upwards from 50% among individuals over the age of 50 (Mueller et al., 2018). Evidence supports the contribution of pet ownership to some aspects of healthy aging (Friedmann, 2019; Friedmann & Gee, 2018), including improved cardiovascular health and decreased mortality (Gee & Mueller, 2019; Levine et al., 2013).

The biopsychosocial model provides potential mechanisms for the impact of human–animal interaction on human health. In this conceptualization, biological, psychological, and social variables affect each other and integrate into health. Human–animal interaction can be conceptualized as a source of enhancement in each variable group (Friedmann, 2019; Gee et al., 2021). The psychological and biological effects of interacting with an animal are often closely interwoven; human–animal interaction leads to reductions in a variety of biological stress indicators such as cortisol, blood pressure, and heart rate. Likewise, the psychological and social variable groups are tightly interwoven; human–animal interaction may provide a source of social support or attachment, which in turn may contribute to lower stress and better psychological health (Friedmann, 2019).

Pet ownership is theorized to support physical health and thereby promote healthy aging (Friedmann & Gee, 2018; Gee & Mueller, 2019). Research links pet ownership with better health outcomes in older adults, particularly those with chronic health conditions, with the strongest evidence from studies of individuals with heart disease (Gee & Mueller, 2019; Levine et al., 2013). The first evidence of the association of pet ownership with physical health was from a study of patients hospitalized with coronary heart disease (Friedmann et al., 1980). That and follow-up studies confirmed that the association of pet ownership with survival was independent of disease severity and social support (Friedmann & Thomas, 1995; Friedmann et al., 2011). The evidence-base for the link between pet ownership and cardiac health in older adults is not consistently positive. In one study examining patients hospitalized for acute cardiac symptoms, pet ownership, specifically cat ownership, was related to the composite outcome of mortality or readmission (Parker et al., 2010). One of the ways pet ownership is suggested as a mechanism for improved cardiovascular health is to encourage physical exercise, and this function is expected to be limited to dog owners, specifically those who walk their dogs (Levine et al., 2013). The American Heart Association validated the idea that pet ownership supported physical activity when they conducted and published a review of the existing evidence, carefully weighing all the results, and issued a statement that pet ownership, particularly dog ownership, likely plays a causal role in reducing the risk of cardiovascular disease. Much of that benefit was attributed to physical activity with the dog (Levine et al., 2013). Another frequently suggested mechanism is that pets decrease stress and stress responses, which decrease cardiovascular-related risk. A repeated measures study using ecological momentary assessment of blood pressure during their daily lives individuals with pre- to mild-hypertension found that stress was lower when they were in the presence of their pets in their homes compared with when their pets were not present (Friedmann et al., 2013).

Cross-sectional studies of the general older adult population also link any current pet ownership to positive health outcomes. Community-residing older adults who owned dogs or cats in the past, but not current owners, experienced reduced odds of becoming frail over 2 years than those who never owned a dog (Taniguchi et al., 2019). Several cross-sectional studies of older adults suggest that dog walking is associated with overall greater physical activity (Coleman et al., 2008; Dall et al., 2017; Mein & Grant, 2018; Thorpe et al., 2006). For example, comparing community-dwelling older adult dog owners with matched nonowners, dog-owners averaged 22 min more of walking time, walked 2,760 more steps, and had fewer prolonged sedentary events per day than nonowners (Dall et al., 2017). Dog walkers on average walk their dogs 160 min per week divided into an average of four walks (Christian et al., 2013). Overall community-dwelling older adult dog owners were 12% more active than nonowners after controlling for potential confounders (Feng et al., 2014). Other large population-based studies of older adults indicated that individuals with a history of pet ownership had greater motor fitness and completed more walking activities than those with no history of pet ownership (Taniguchi et al., 2018). A small prospective observational study of older adults revealed that heart rate variability, a stress indicator, was better (higher) when walking with a dog than when walking alone (Motooka et al., 2006). Older adults who walk their dogs may also experience other benefits, such as greater community engagement (Toohey et al., 2013; Wood et al., 2005), positive feelings about their neighborhoods (Mein & Grant, 2018), and social benefits (Antonacopoulos & Pychyl, 2014; McNicholas & Collis, 2000; Taniguchi et al., 2018; Wells, 2004).

Longitudinal studies have an advantage over cross-sectional studies in establishing a temporal relationship between exposure and outcomes. In this study, we use longitudinal data to examine the associations of pet ownership with maintaining physical function and leisure time physical activity in the generally healthy community-dwelling older adult participants in the Baltimore Longitudinal Study of Aging (BLSA).

This study provides an opportunity to examine this speculation. We expected deterioration in physical function and leisure time physical activity as participants aged. We hypothesized that pet ownership, as well as dog ownership, and cat ownership separately, are associated with maintaining physical function and leisure time physical activity and that among dog owners, dog walking is associated with maintaining physical function and leisure time physical activity for generally healthy community-dwelling older adults participating in the BLSA.

## Design

The study used a cohort design with health data obtained in the BLSA, an ongoing National Institute on Aging (NIA) Intramural Research Program-funded cohort study. The BLSA is America’s longest-running scientific study of human aging. Started in 1958, BLSA is a longitudinal observational study that addresses critical questions about normal and pathological age-related change. BLSA uses a standardized battery of surveys and tests to measure cognitive and physical changes associated with aging in real-time. Each BLSA visit consists of three consecutive days of testing. Participants who are 20–60 years old complete BLSA visits every 4 years; those 60–79 years old complete them every 2 years; and those 80 years and older complete them annually. Following IRB approval by the National Institutes of Health Intramural Research Program Institutional Review Board, a group of pet ownership-related questions, including a 10-year pet ownership history, were added to the battery of surveys completed during visits starting in March 2017. The BLSA visit that included the individual’s first assessment of human–animal interaction is termed the survey visit. Individuals aged 50 and older at the survey visit and with two or more BLSA visits were included in the study.

**Figure 1.**
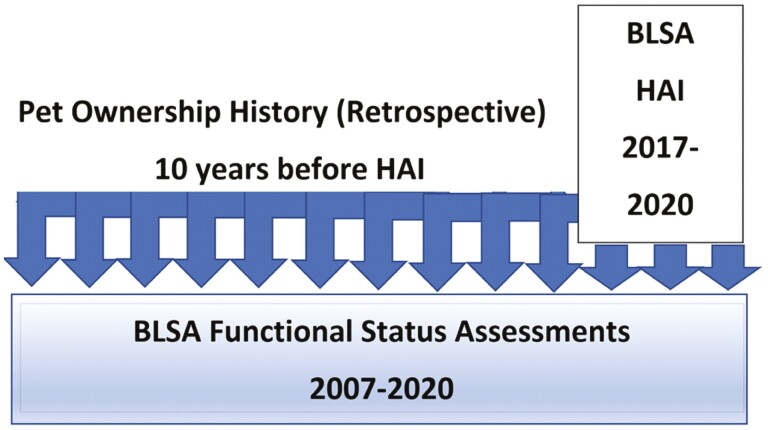
Human–animal interaction (HAI) assessment module and pet ownership history assessment in the context of the Baltimore Longitudinal Study of Aging (BLSA). The survey visit is the first BLSA visit with a human–animal interaction assessment (March 2017 to March 2020). The index visit is the first BLSA visit in the 10 years prior to the survey visit.

Participants included in this study had been completing visits to the BLSA for varying times prior to the survey visit. They also continued to attend their regularly scheduled BLSA visits for up to 3 years after the survey visit. Thus, the BLSA participants in this study attended BLSA visits and provided functional data at specified BLSA intervals over a period of 1–13 years, with a mean follow-up of 7.5 years (standard deviation [*SD*] = 3.6). Pet ownership data from the pet ownership history component of the human–animal interaction assessment were associated with each BLSA visit for the 10 years prior to the survey visit and for any visits during the subsequent 3 years of the study. The first BLSA visit associated with pet ownership data is termed the index visit (see Figure 1).

## Participants

A total of 637 participants in the BLSA were aged 50–100 years (*M* = 75.1, *SD* = 10.15). The sample was predominantly white (66.98%) with smaller proportions of Black (28.12%), Asian (1.26%), and Hawaiian/Pacific Islanders (0.32%) represented. For analyses, Asians and Hawaiian/Pacific Islanders were grouped with Whites. The sample was relatively equally distributed between male (44.11%) and female (55.89%). A large majority of the sample held postgraduate degrees (67.03%). The sample was also largely married or partnered (62.20%) and living with at least one other person (70.96%), and in a single-family home (78.74%). Only 16.61% of the sample had an income less than $50,000 per year. Less than half of the sample currently work (32.60%), while 54.17% volunteered.

Among the 637 participants who completed the survey visit, 185 (29.0%) owned pets, 112 (17.58%) owned cats and 95 (14.91%) owned dogs; 22 (7.33%) owned either other species of pets only or a combination of multiple species of pets including cats or dogs. Among dog owners 58 (69.05%) indicated they walked their dogs. There were notable differences in race and housing type between pet owners and nonpet owners, summarized in Table 1. Pet ownership was significantly more common among whites than blacks and among those who resided in single-family homes than those who did not (*p*’s < .001).

**Table 1.** Demographic and Pet Ownership Characteristics of Respondents at the Baltimore Longitudinal Study of Aging Study Survey Visit When Participants First Completed the Human–Animal Interaction Questionnaire

Characteristic	Overall (*n* = 637)				Nonpet owner (*n* = 452)				Pet owner (*n* = 185)				Test statistic	*p*
	*N*	%	*M*	*SD*	*N*	%	*M*	*SD*	*N*	%	*M*	*SD*		
Dog owner	112	17.58			0	n/a			112	60.54				
Cat owner	95	14.91			0	n/a			95	51.35				
Age in years^a^	75.09	10.15			76.76	9.67			71.01	10.15			6.71	**<.001**
Black^b^	178	27.94			146	32.30			32	17.30			14.68	**<.001**
Female	356	55.89			257	56.86			99	53.51			0.60	.440
<College Graduate^b^	80	12.62			65	14.44			15	8.15			5.03	.081
Married or partnered^b^	395	62.20			263	58.31			132	71.74			10.02	**.002**
Live alone^b^	176	29.04			143	33.41			33	18.54			13.49	**<.001**
Live in single family house^c^	500	78.74			328	72.73			172	93.48			33.62	**<.001**
Family income exceeds $50,000^b^	527	83.39			359	83.10			168	92.31			8.92	**.003**
Currently works^b^	207	32.60			131	29.11			76	41.30			8.83	**.003**
Currently volunteers^b^	344	54.17			241	53.56			103	55.98			0.31	.578
Body mass index^a^			26.98	4.81			27.10	4.87			26.54	4.66	0.96	.339
Obese^b^	148	23.23			107	23.67			41	22.16			0.17	.682
Comorbidities^a^			1.66	1.48			1.73	1.49			1.50	1.46	1.84	.066
Follow-up years^a^			6.84	3.57			7.01	3.34			6.43	3.73	1.91	.057
400-m Walk time (wln)^d^			289.38	68.17			297.7	70.01			270	59.44		**<.001**
Usual speed^a^			1.12	0.24			1.09	0.24			1.20	0.24	−5.06	**<.001**
Rapid speed^a^			1.66	0.38			1.61	0.37			1.78	0.38	−4.97	**<.001**
Physical performance^a^			2.53	0.62			2.45	0.63			2.72	0.57	−4.89	**<.001**
Physical well-being(rln)^d^^,^^e^			51.67	7.59			51.26	7.74			52.65	7.15		**.005**
Leisure activity^d^			3,867.68	3,788.39			3,450.30	3,394.00			4,868.01	4,451.70		**<.001**
Leisure walking (ln)^a^			3.31	3.56			3.04	3.66			3.95	3.21	−2.88	**.004**
Brisk exercise (ln)^a^			−0.49	4.60			−0.75	4.55			0.14	4.65	−2.18	**.030**
Weekly exercise (ln)^a^			1.17	4.54			1.005	4.55			1.56	4.48	−1.37	.171
Recommended exercise^b^	149	24.55			101	23.60			48	26.82			0.71	.401

*Notes*: 400-m Walk time = seconds to walk 400 meters; usual speed = usual gait speed (m/s); rapid speed = rapid gait speed (m/s); physical performance = physical performance battery score; physical well-being = SF-12 physical function component score; leisure activity = total leisure time physical activity (calories) per week; leisure walking = minutes of leisure walking per week; brisk exercise = minutes of “brisk” or exercise related walking per week; weekly exercise = minutes spent exercising per week; recommended exercise = dichotomous indicator for achieved recommended 150 min of moderate intensity exercise per week; (ln) = natural log transformed; (rln) = reflected and natural log transformed; (wln) = winsorized and natural log transformed. Bold is used to highlight significance *p* <.05.

^a^Student’s *t*-test.

^b^Chi-Square test.

^c^Fischer’s exact test.

^d^Wilcoxon rank sum test.

^e^Score reflected, lower is better.

Annual pet ownership data were obtained retrospectively during the survey visit for the preceding 10 years. These pet ownership data were mapped onto the BLSA physical assessment visits over that same period. The earliest occasion when pet ownership data could be associated with a BLSA assessment was used to produce the index visit data. Pet ownership data were associated with each BLSA physical assessment within the last 10 years as well as the 3 years of the study after the survey visit.

The characteristics of the participants at the index visit are included in Supplementary Table 1. These data are baseline data for all further analyses. Average age at index visit was 68.25 years (*SD* = 9.64). The participants were generally in good health; participants’ mean number of comorbidities was 0.95 (*SD* = 1.17). Only 20.88% of the participants were obese.

At the index visit, 188 (29.51%) of the 637 participants owned pets; 67 (10.52%) owned cats, and 84 (13.19%) owned dogs. Other pet owners did not indicate the types of pets they owned. Pet owners were significantly younger (*p* < .001) and had fewer comorbidities (*p* = .030) than nonowners. There were notable differences in race and housing type between pet owners and nonpet owners, like those reported for the survey visit.

There were no significant differences in the demographic characteristics of cat owners and dog owners at the index visit. Dog owners were not significantly younger and did not have a better initial physical function, although they did have significantly fewer comorbidities (*p* = .039) than cat owners. At the index visit, individuals who owned dogs but not cats (dog owners exclusively) and individuals who owned cats but not dogs (cat owners exclusively) shared similar demographic characteristics (Table 2). Dog owners exclusively (*n* = 79) were 65.52 years old on average, while cat owners exclusively (*n* = 62) were 66.60 years old at the time of initial assessment. Most cat owners exclusively and dog owners exclusively were white (83.87% and 78.67%, respectively) and predominantly female (*n* = 77, 54.61%). Both cat owners exclusively (77.42%) and dog owners exclusively (70.51%) had high levels of postgraduate education. Cat owners exclusively had significantly more comorbidities (*M* = 1.05, *SD* = 1.25) than dog owners exclusively (*M* = 0.75, *SD* = 1.15, *p* = .041). Cat owners exclusively were followed longer in the study (*M* = 7.71, *SD* = 3.38) than dog owners exclusively (*M* = 6.16, *SD* = 4.29, *t* = −2.33, *p* = .02). None of the physical function variables differed significantly between cat owners exclusively and dog owners exclusively.

**Table 2.** Demographic and Pet Ownership Characteristics of Pet Owners Comparing Those That Own Cats Exclusively (Without Owning Dogs) to Those Who Own Dogs Exclusively (Without Owning Cats) Index Visit

Characteristic	Overall (*n* = 141)				Cat owners (*n* = 62)				Dog owner(*n* = 79)				Test Statistic	*p*
	*N*	%	*M*	*SD*	*N*	%	*M*	*SD*	*N*	%	*M*	*SD*		
Age in years^a^			65.99	9.22			66.60	8.58			65.52	9.72	−0.69	.489
Black^b^	26	18.44			10	16.13			16	20.25			0.39	.531
Female^b^	77	54.61			37	59.68			40	50.63			1.15	.284
<College graduate^c^	8	5.71			4	6.45			4	5.13				.441
Married or partnered^b^	108	77.14			49	79.03			59	75.64			0.23	.635
Lives alone^b^	25	17.86			9	14.52			16	20.51			4.41	.221
Single family housing^c^	132	94.96			60	98.36			72	92.31				.135
Family income exceeds $50,000^b^	120	86.96			53	86.89			67	87.01			0.001	.982
Currently works^b^	84	60.00			35	56.45			49	62.82			0.58	.445
Currently volunteers^b^	79	57.25			39	63.93			40	51.95			2.00	.158
Body mass index^a^			26.49	4.58			25.81	4.28			27.03	4.76	1.57	.118
Obese^b^	24	17.02			6	9.68			18	22.78			4.23	**.040**
Comorbidities^d^			0.80	1.20			1.05	1.25			0.75	1.15		**.041**
Follow-up years^a^			6.84	3.98			7.71	3.38			6.16	4.29	−2.33	**.021**
400-m Walk time (wln)^d^			251.94	48.53			256.9	55.77			247.79	41.52		.376
Usual speed^a^			1.23	0.22			1.22	0.20			1.23	0.23	0.23	.818
Rapid speed^a^			1.88	0.35			1.87	0.34			1.89	0.36	0.36	.722
Physical performance^d^			2.74	0.47			2.71	0.38			2.77	0.53		.240
Physical well-being (rln)^d^^,^^e^			53.61	6.02			53.17	6.52			53.96	5.60		.657
Leisure activity^a^			6,433.07	4,322.20			5,023.85	4,027.53			6,713.54	4,954.53	1.97	.051
Leisure walking (ln)^a^			3.88	3.12			4.38	2.96			3.26	3.23	2.12	**.036**
Brisk exercise (ln)^a^			0.67	4.53			1.05	4.59			0.19	4.45	1.12	.26
Weekly exercise (ln)^a^			1.82	4.33			2.22	4.28			1.33	4.37	1.2	.231
Recommended exercise^b^	41	29.29			26	33.33			15	24.19			1.39	.238

*Notes*: 400-m Walk time = seconds to walk 400 meters; usual speed = usual gait speed (m/s); rapid speed = rapid gait speed (m/s); physical performance = physical performance battery score; physical well-being = SF-12 physical function component score; leisure activity = total leisure time physical activity (calories) per week; leisure walking = minutes of leisure walking per week; brisk exercise = minutes of “brisk” or exercise related walking per week; weekly exercise = minutes spent exercising per week; recommended exercise = dichotomous indicator for achieved recommended 150 min of moderate intensity exercise per week; (ln) = natural log transformed; (rln) = reflected and natural log transformed; (wln) = winsorized and natural log transformed. Bold is used to highlight significance *p* <.05.

^a^Student’s *t*-test.

^b^Chi-square test.

^c^Fischer’s exact test.

^d^Wilcoxon rank sum test.

^e^Score reflected, lower is better.

Among the 73 dog owners who indicated whether they walked their dogs at the survey visit, 58 (79.5%) indicated they walked their dogs. Index visit characteristics of dog owners who walked and did not walk their dogs are included in Table 3. The demographics of the two groups did not differ, but health status did. Dog walkers had significantly lower impairment in activities of daily living and lower triglycerides. Comorbidities did not differ significantly between dog walkers and nonwalkers.

**Table 3.** Demographic and Pet Ownership Characteristics of Dog Owners (*n* = 73) Who Walk and Do Not Walk Their Dogs

Characteristic	Overall (*n* = 73)				Dog walkers (*n* = 58)				Nondog walkers (*n* = 15)				Test statistic	*p*
	*N*	%	*M*	*SD*	*N*	%	*M*	*SD*	*N*	%	*M*	*SD*		
Age in years^a^			64.48	9.89			64.11	9.36			65.92	12.00	0.63	.532
Black^b^	12	16.44			10	17.24			2	13.33				1.000
Female^b^	38	52.05			27	46.55			11	73.33			3.43	.064
<College graduate^c^	3	4.17			1	1.75			2	13.33				.541
Married or partnered^b^	55	76.39			41	71.93			14	93.33				.100
Lives alone^b^	14	19.72			10	17.86			4	26.67				.475
Single family housing^c^	66	91.67			53	92.98			13	86.67				.598
Family income exceeds $50,000^b^	62	87.32			48	84.21			14	100				.189
Currently works^b^	47	65.28			38	66.67			9	60.00			0.23	.629
Currently volunteers^b^	33	46.48			26	46.43			7	46.67			0.001	.987
Body mass index^a^			26.72	4.51			26.51	3.79			27.54	6.72	0.57	.575
Obese^b^	15	20.55			11	18.97			4	26.67			0.43	.511
Comorbidities^d^			0.68	1.17			0.50	0.80			1.40	1.92		.175
Follow-up years^a^			5.46	4.31			5.44	4.41			5.51	4.06	0.06	.955
Attachment^a^			65.70	11.57			66.39	11.34			63.21	12.48	−0.94	.349
400-m Walk time (wln)^d^			242.92	38.85			241.41	35.62			250.45	53.68		.973
Usual speed^a^			1.25	0.23			1.24	0.19			1.28	0.34	0.39	.700
Rapid speed^a^			1.91	0.36			1.91	0.30			1.94	0.53	0.26	.795
Physical performance^d^			2.84	0.54			2.84	0.42			2.82	0.90		.615
Physical well-being (rln)^d^^,^^e^			54.16	5.19			55.05	3.29			50.81	8.82		.0518
Leisure activity^a^			6,602.87	4,895.25			6,489.20	4,193.20			5,518.70	4,210.60	−0.78	.441
Leisure walking (ln)^a^			4.45	2.90			2.47	4.74			4.93	2.02	−3.01	**.004**
Brisk exercise (ln)^a^			0.86	4.61			-0.09	5.07			1.11	4.50	−0.89	.376
Weekly exercise (ln)^a^			2.11	4.32			0.63	5.12			2.50	4.04	−1.51	.136
Recommended exercise^b^	23	31.94			4	26.67			19	33.33			0.24	.622

*Notes*: 400-m Walk time = seconds to walk 400 meters; usual speed = usual gait speed (m/s); rapid speed = rapid gait speed (m/s); physical performance = physical performance battery score; physical well-being = SF-12 physical function component score; leisure activity = total leisure time physical activity (calories) per week; leisure walking = minutes of leisure walking per week; brisk exercise = minutes of “brisk” or exercise related walking per week; weekly exercise = minutes spent exercising per week; recommended exercise = dichotomous indicator for achieved recommended 150 min of moderate intensity exercise per week; (ln) = natural log transformed; (rln) = reflected and natural log transformed; (wln) = winsorized and natural log transformed. Bold is used to highlight significance *p* <.05.

^a^Student’s *t*-test.

^b^Chi-square test.

^c^Fischer’s exact test.

^d^Wilcoxon rank sum test.

^e^Score reflected, lower is better.

## Measures

### Pet Ownership Measures

Pet ownership-related variables were assessed using multiple sources, including a 10-year pet ownership history questionnaire and the NIA Health and Retirement Study (HRS) pet ownership interaction module (National Center for Health Statistics, 2020; NIA, 2012). The HRS pet ownership module queried individuals who owned dogs about dog walking behavior, including whether they walked their dog, how frequently they walked their dog, and the average duration of walks (Friedmann et al., 2020).

### Physical Function Measures

The BLSA physical function measures include measures of cardiorespiratory fitness (400-m walk time), lower body strength (usual and rapid gait speed), overall physical performance (physical performance), participants’ perceptions of their physical function (physical well-being), and leisure time physical activities (leisure activity, leisure walking, brisk exercise, and recommended exercise).

#### Cardiorespiratory fitness

Cardiorespiratory fitness was assessed using 400-m walk time Participants walked 10 laps around a 40-m course on a carpeted interior corridor while accompanied by a study member counting the laps. They were instructed to do so as quickly as possible. Each lap time was recorded. Participant scores were calculated based on the use of assistive devices and their ability to complete the distance either with or without a walking aid (Simonsick et al., 2001a). Participants received standard encouragement and were told the number of laps remaining (Martinez-Amezcua et al., 2021). Time to complete the 400-m walk has previously been associated with both physical activity level and cardiovascular disease, with lower walk time predicting increased physical activity level and decreased cardiovascular disease (Trombetti et al., 2016; Winger et al., 2020). Four hundred meter walk time is validated as a measure of physical fitness by a negative correlation (−0.79) with peak VO2 (Simonsick et al., 2006). Lower time indicates better function.

#### Lower body strength

Lower body strength was assessed over a 6-m indoor carpeted course with participants walking at their usual walking speed (usual gait speed) for 6 m twice and then at their maximum walking speed (rapid gait speed) for 6 m twice. Time for each walk was measured with a stopwatch to the hundredth of a second. The time for the fastest of the two maximum speed walks was divided into six and provided the rapid gait speed (m/s; Schrack et al., 2012). This measure is validated in older adults by treadmill validation of an association with maximum oxygen consumption (Simonsick et al., 2006). Higher scores reflect faster gait in meters per second. The ideal usual gait speed is ≥1.0 m/s, however, certain daily activities may necessitate faster speeds (Sprague et al., 2021).

#### Overall physical performance

The Healthy Aging and Body Composition Study physical performance battery was used to assess the overall physical function of each participant. The score was derived as a composite of the four components from the Short Physical Performance Battery: usual gait speed as described earlier, time to stand-up from and sit back down on an armless chair five times, ability to hold three progressively more challenging balance-related stances (semitandem, full-tandem and single-leg for up to 30 s) each, and ability and time to walk a narrow (20 cm wide) 6-m course. For the narrow walk, three attempts were completed with the fastest successful walk time used as the score; having three failures was scored as zero. Each test was scored on a ratio scale with maximal performance as the denominator and actual performance as the numerator. Maximal performance for usual gait speed and the narrow walk was 2.0 m/s, for five chair stands was 5 s, and for the standing balance test was 90 s. For the narrow walk, three attempts were permitted to walk without stepping on or outside the limits of the course more than twice. Any performance exceeding the defined maximum was assigned a score of 1.0 for that test. The physical performance score constitutes the sum of these four ratio scores for a maximum total of 4.0 (Simonsick et al., 2016; Simonsick et al., 2001b). Higher values on this well-validated measure (Simonsick et al., 2006) indicate better physical performance.

#### Physical well-being

The Medical Outcomes Study Short Form-12 (SF-12) was used to provide individuals’ assessments of their own well-being. It consists of 12 self-rated items that capture eight domains of self-rated health that generate physical component summary (physical well-being) and mental component summary (mental well-being) scores calibrated to population norms (Ware, 2005; Ware et al., 1996). According to the Centre for Health Service Development (Marosszeky & Sansoni, 2005), a meta-analysis instrument review revealed SF-12 to be a psychometrically sound tool with test–retest reliability for the physical well-being score of 0.89; higher scores indicate greater physical well-being (Frantzen et al., 2021; Marosszeky & Sansoni, 2005)

### Leisure Time Physical Activity Measures

Five measures of weekly leisure time physical activity were calculated: total leisure time physical activity (leisure activity), time spent walking (leisure walking), minutes spent in “brisk” or exercise-related walking (brisk exercise), minutes spent exercising (weekly exercise), and a dichotomous variable (recommended exercise) indicating whether the participant got the recommended 150 min of weekly moderate-intensity exercise (Brach et al., 2004). Leisure time physical activity was determined using a standardized questionnaire designed specifically for the Health ABC study, modeled from commonly used physical activity assessments, including the leisure time physical activity questionnaire. Participants were asked if they participated in specific activities at least 10 times in the past 12 months; if so, whether they had done them in the past 7 days; and if so, the amount of time spent doing each. They were also asked to name other physical activities they participated in and answer similar questions about them. Physical activity amounts were calculated based on activities in the past 7 days and metabolic equivalent values of the tasks (Brach et al., 2004). Higher values indicate greater leisure time physical activity.

### Covariates

Both increasing age and comorbidities are generally associated with diminished physical function. These variables have the potential to confound the relationship of pet ownership with physical function because pet owners were younger and had fewer comorbidities than nonowners. Therefore, age and comorbidities were added as predictors in analyses of changes in physical health with aging. Age and comorbidities at the first time for which both pet ownership and BLSA functional data are available were used as covariates where indicated. A summary of physical comorbidities at the same time was also used as a covariate where indicated. Comorbidities scores were calculated as the sum of the number of eight conditions that the respondents reported (Friedmann et al., 2020). Conditions assessed were heart disease (including angina, myocardial infarction, heart failure, angioplasty, and coronary artery bypass graft), diabetes, pulmonary disease, cerebral vascular disease, lower extremity arthritis, lower extremity pain, minor functional difficulty, and exertional pain while walking.

## Statistics

Descriptive statistics were used to portray the participants and their index and survey visit characteristics. Characteristics of pet owners and nonowners at both index and survey visits were compared using *t*-tests for normally distributed continuous variables, Wilcoxon rank sum test for nonnormally distributed continuous variables, chi-square tests for categorical variables, and Fisher’s exact tests for categorical variables with expected cell frequencies lower than five. Differences in index visit physical function between cat and dog owners, and in dog owners who walked their dogs at survey visit and those who did not were examined in a similar manner. Prior to multivariable analysis, data were cleaned and examined for outliers and normality. Four-hundred-meter walk time, leisure walking, brisk exercise, and weekly exercise were log-transformed and physical well-being was reflected, and natural log-transformed. Unconditional intraclass correlations indicated considerable dependence ranging from 0.38 (400-m walk time) to 0.75 (physical performance; Supplementary Table 2).

A linear mixed models (LMM) approach was used to examine changes in physical function and leisure time physical activities and compare changes by pet ownership status, except a generalized logistic model was used for recommended exercise, which had a dichotomous outcome. Pet ownership associated with each BLSA assessment was used as a time-varying covariate, unless noted otherwise. Thus, pet ownership at each data collection point was considered in all analyses except when pet ownership at index visit is specifically stated. Linear, or generalized linear, mixed models were used to examine changes in outcomes over up to 13 years (*M* = 7.5, *SD* = 3.6). Separate models were used to examine the association of pet ownership, dog ownership, and cat ownership to change in each outcome over time. Subsequently, these analyses were rerun to include age and comorbidities as covariates. In addition, LMMs that simultaneously included cat ownership, dog ownership, and the covariates were used to examine the independent associations of cat and dog ownership to longitudinal changes in each physical function and leisure time physical activity. We then ran LMM adjusting for covariates that tested the associations of cat ownership versus dog ownership to longitudinal changes in physical function and leisure time physical activity. A similar LMM approach was used to examine differences in both overall physical function/leisure time physical activities and changes in physical function/leisure time physical activity between dog owners who walked their dogs at the survey visit and those who did not. Dog walking analyses modeled the effect of this first dog walking status (time-invariant). Power was adequate (>80%) with a medium effect size for the changes in outcomes according to pet ownership, dog ownership, and cat ownership, but not for the smaller samples comparing dog owners who walked and did not walk their dogs. We calculated Cohen’s *d* for the significant interactions of aging and pet ownership status. We calculated the difference in changes in outcomes over time between pet owners and nonowners and divided this by the raw baseline outcome standard deviation.

## Results

At the index human–animal interaction assessment, 185 (29.04%) participants owned pets; 95 (14.91%) owned cats, and 112 (17.58%) owned dogs. Fewer than 5% of the participants owned a pet other than a cat or dog (Friedmann et al., 2020). Index visit physical function of pet owners were compared with those of nonowners (Supplementary Table 1). At the index visit, pet owners took significantly less time to walk 400 m, indicating better cardiorespiratory fitness, had faster usual and rapid gait speed, better overall physical performance, and reported more total leisure time physical activity but no other measures of leisure time physical activity, than nonowners. There were no significant differences in physical well-being between pet owners and nonowners.

### Changes in Outcomes With Aging

As hypothesized, all physical function outcomes declined over time, *p*’s < .001 (Supplementary Table 3).

### Association of Pet Ownership With Changes in Outcomes With Aging

**Figure 2.**
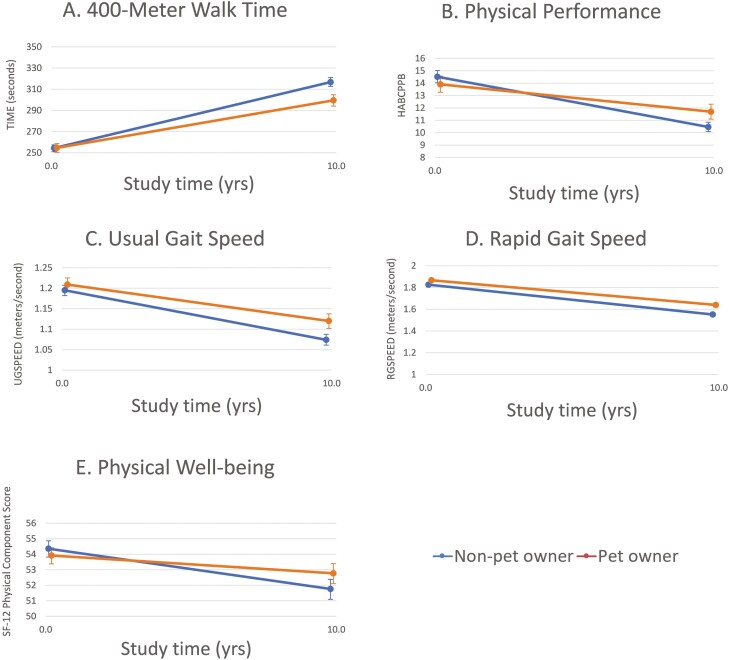
Changes in five measures of physical function according to pet ownership status with 10 years of aging, estimates based on linear mixed models that include age and comorbidities as covariates.

Pet ownership was associated with less decline in most physical functional domains, but not in leisure time physical activities, after controlling for age and comorbidities (Table 4). Pet owners experienced significantly smaller increases in time to walk 400 m (Figure 2A, effect size [ES] = −0.40), decreases in physical performance score (Figure 2B, ES = 0.35), usual and rapid gait speed (Figures 2C and D, ES = 0.15, 0.13), and physical well-being than nonowners (Figure 2E, ES = −0.25).

**Table 4.** Changes in Physical Function Variables With Aging According to Pet Ownership, Dog Ownership, and Cat Ownership, Adjusted for Age and Comorbidity (*n* = 637)

Outcome	Interaction	With	Follow-up	Interaction	With	Follow-up	Interaction	With	Follow-up
	PO est	PO *se*	PO *p*	DO est	DO *se*	DO *p*	CO est	CO *se*	CO *p*
400-m Walk time (wln)	−0.006	0.001	**<.001**	−0.002	0.001	.113	−0.005	0.001	**<.001**
Physical performance	0.015	0.003	**<.001**	0.009	0.004	**.046**	0.015	0.004	**<.001**
Usual speed	0.003	0.002	**.032**	0.003	0.002	.244	0.002	0.002	.346
Rapid speed	0.005	0.002	**.030**	0.008	0.003	**.015**	0.002	0.003	.507
Physical well-being (rln)^a^	−0.008	0.003	**.002**	−0.002	0.004	.501	−0.009	0.003	**.009**
Leisure activity	−21.594	25.549	.398	−36.534	36.648	.319	−3.633	35.162	.918
Leisure walking (ln)	0.040	0.031	.195	0.056	0.044	.202	−0.010	0.042	.820
Brisk exercise (ln)	−0.015	0.041	.717	−0.057	0.058	.331	0.052	0.057	.361
Weekly exercise (ln)	0.019	0.039	.630	−0.042	0.056	.447	0.092	0.054	.092
Recommended exercise	−0.005	0.033	.888	−0.036	0.044	.821	−0.035	0.047	.455

*Notes*: PO = pet ownership, nonownership is reference category; DO = dog ownership, nonownership is reference category; CO = cat ownership, nonownership is reference category; est = parameter estimate; *se* = standard error; 400-m Walk time = seconds to walk 400 meters; usual speed = usual gait speed (m/s); rapid speed = rapid gait speed (m/s); physical performance = physical performance battery score; physical well-being = SF-12 physical function component score; leisure activity = total leisure time physical activity (calories) per week; leisure walking = minutes of leisure walking per week; brisk exercise = minutes of “brisk” or exercise related walking per week; weekly exercise = minutes spent exercising per week; recommended exercise = dichotomous indicator for achieved recommended 150 min of moderate intensity exercise per week; (ln) = natural log transformed; (rln) = reflected and natural log transformed; (wln) = winsorized and natural log transformed. Bold is used to highlight significance *p* <.05.

^a^Score reflected, lower is better.

### Associations of Dog Ownership and Cat Ownership With Changes in Outcomes With Aging

In separate analyses, both dog ownership and cat ownership were associated with less decline in some physical function measures (Table 4). Dog owners experienced significantly less deterioration in overall physical performance (*p* = .046, ES = 0.20) and rapid gait speed (*p* = .015, ES = 0.22) than people who did not own dogs after controlling for age and comorbidities. Cat owners exhibited significantly less deterioration in 400-m walk speed (*p* < .001, ES = −0.36), physical function (*p* < .001, ES = 0.35), and physical well-being (*p* = .009, ES = −0.29) than people who did not own cats after controlling for age and comorbidities. Dog and cat ownership were not associated with changes in leisure time physical activity measures.

Additional analyses examined the independent associations of dog ownership and cat ownership to physical function outcomes by entering both dog ownership and cat ownership into the models simultaneously. They provided similar results (Supplementary Table 4). Sensitivity analysis (Supplementary Table 5) using index visit pet ownership characteristics instead of time-varying pet ownership characteristics at the time of each physical function assessment as predictors produced similar results as those reported in Table 4, with two exceptions. Pet ownership at the index visit was not related to changes in rapid gait speed, and dog ownership at the index visit was not related to changes in overall physical performance.

Analyses comparing changes in the physical function of dog owners exclusively and cat owners exclusively with aging (Table 5) demonstrated no significant difference between the groups. Changes with aging in 400-m walk time, physical performance, usual gait speed, rapid gait speed, physical well-being, and leisure time physical activity did not differ significantly between dog owners exclusively and cat owners exclusively.

**Table 5.** Estimates for Interaction Parameters From Linear Mixed Models Examining the Associations of Dog Ownership Exclusively (Without Cat Ownership) Versus Cat Ownership Exclusively (Without Dog Ownership) and the Association of Dog Walking to Changes in Physical Function Variables With Aging, Adjusted for Age and Comorbidity (*n* = 141, 73, Respectively)

Outcome	Interaction	With	Follow-up	Interaction	With	Follow-up
	DvC est	DvC *se*	DvC *p*	DW est	DW *se*	DW *p*
400-m Walk time(wln)	0.003	0.002	.110	−0.001	0.003	.828
Physical performance	−0.011	0.006	.068	−0.0004	0.010	.968
Usual speed	−0.002	0.003	.543	−0.002	0.005	.660
Rapid speed	0.002	0.005	.645	0.011	0.009	.239
Physical well-being (rln)^a^	0.004	0.005	.481	0.005	0.009	.593
Leisure activity	−15.557	52.786	.768	−16.318	99.130	.869
Leisure walking (ln)	0.102	0.056	.069	−0.265	0.081	.001
Brisk exercise (ln)	−0.069	0.084	.413	−0.071	0.144	.624
Weekly exercise (ln)	−0.111	0.081	.171	−0.045	0.140	.751
Recommended exercise	0.094	0.067	.163	0.094	0.067	.163

*Notes*: DvC = dog vs cat ownership, reference category is cat ownership; DW = dog walkers vs nonwalkers, reference category is nonwalkers; est = parameter estimate; *se* = standard error; 400-m Walk time = seconds to walk 400 meters; usual speed = usual gait speed (m/s); rapid speed = rapid gait speed (m/s); physical performance = physical performance battery score; physical well-being = SF-12 physical function component score; leisure activity = total leisure time physical activity (calories) per week; leisure walking = minutes of leisure walking per week; brisk exercise = minutes of “brisk” or exercise related walking per week; weekly exercise = minutes spent exercising per week, Recommended exercise = dichotomous indicator for achieved recommended 150 min of moderate intensity exercise per week, (ln) = natural log transformed, (rln) = reflected and natural log transformed, (wln) = winsorized and natural log transformed.

^a^Score reflected, lower is better.

### Dog Walking and Change in Outcomes With Aging

There were no significant differences in changes in physical function or and most leisure time physical activity variables with aging between dog owners who walked their dogs and those who did not (Table 5). However, time spent in leisure walking changed differently for dog owners who walked and did not walk their dogs. Leisure time walking was greater for dog owners who walked their dogs at index visit. Dog-walkers’ leisure time walking decreased over time, while nondog-walkers’ leisure time walking increased (*p* = .001, es = −0.74). At 10 years, dog walkers’ and nonwalkers’ leisure time walking did not differ significantly (point estimate = 4.374, 95% confidence interval [CI] 3.770 to 4.979, point estimate = 4.496, 95% CI 3.319 to 5.619, *p* = .886).

## Discussion

The results of this study indicate that pet ownership is associated with better-preserved physical function in community-dwelling older adults. This work provides an important extension of previous cross-sectional evidence discussed in the introduction by providing a longitudinal perspective on pet ownership and physical function. It establishes clear evidence that pet ownership is associated with slower deterioration over time in physical functioning among older adults. Of note, the study includes longitudinal objective measures of physical function as well as the more typical questionnaire-based assessments (Koohsari et al., 2020). In the current study, pet ownership was related to less deterioration in longitudinal objective measures of physical function, but not to changes in leisure time physical activity assessed with questionnaires.

The study did not produce evidence that dog ownership or cat ownership was superior in relationship to maintenance of physical function in older adults. This suggests that aspects other than the physical activity associated with dog ownership are associated with the maintenance of physical function. We can speculate that the need to move about and care for any animal could be a helpful impetus for physical activity that promotes function. Similarly, dog walking was not associated with different trajectories of change in objective measures of physical function. A possible explanation is that dog walking might not add to total walking; the same people who walk with the dog might also perform equal activities to maintain physical function with or without it. This conjecture is supported by the finding that by the end of the study, when fewer individuals owned pets, total walking did not differ significantly between those who walked and did not walk their dogs. Of note, the comparison of dog walkers with dog owners who do not walk their dogs lacks power due to small sample sizes and requires further replication.

In the current study, we controlled for age to address the staggered entry of individuals into the study because older adults’ physical function does decline over time, and it declines faster with age. It is important that the results of this study not be generalized to a younger population, where the physical function may not deteriorate, and the relationship of pet ownership to health may be different. In a population-based study of Australian adolescents, neither pet ownership nor time spent caring for the pet were related to health or well-being (Mathers et al., 2010). It also is possible that time spent with a pet is a factor in how pet ownership is related to health. In the aforementioned study, despite having high rates of pet ownership, adolescents spent little time with their pets (Mathers et al., 2010). Older adults may spend more time with their pets than younger adults or adolescents who spend more time in other leisure time physical activities.

In the current study, we also controlled for comorbidities. The comorbidity index included minor difficulties in physical function. Including this variable may over-adjust and thus obscure some pet ownership or dog walking-related differences in changes in physical function over time.

The findings in this study confirm the importance of using a longitudinal approach to examine of the relationship of exposure and outcomes as well as controlling for confounders. In bivariate cross-sectional analysis, all outcomes except recommended physical activity and high-intensity exercise minutes were significantly related to pet ownership. In contrast, in the longitudinal analyses that controlled for age and comorbidities, changes in all physical function variables were related to pet ownership, but changes in leisure time physical activity were not. This finding suggests that leisure time physical activity would not change whether people kept pets or did not.

In the current study, dog ownership and cat ownership both made independent associations to longitudinal changes in physical function in the same direction but not to leisure time physical activity. The results of this study indicate that dog ownership and cat ownership are related to less deterioration in physical functioning as older adults age. The finding that dog ownership was not related to higher intensity leisure time physical activity is consistent with evidence that that owning a dog was not associated with medium-to-high-intensity physical activities (Koohsari et al., 2020). This implies that the type of person who owns a dog would do more physical activity even if they did not have a pet. No previous studies of which we are aware evaluate the relationship of cat ownership to leisure time physical activity.

Any benefit of dog ownership for older adults would be particularly important for individuals who are socially isolated (Carr et al., 2021). A recent population-based cross-sectional study of 9,856 community-dwelling older adults in Japan found that socially isolated older adults who never owned a dog were more likely to report lower psychological health than current or past socially isolated dog owners (Ikeuchi et al., 2021). Dog ownership may increase opportunities for socially isolated older adults to engage in physical as well as social activities, potentially improving both function and psychosocial health in this population. It is important to recognize that individuals with more impaired ADLs who live alone will have difficulty caring for a pet, especially a dog, without outside assistance. The current study addressed a healthy population, and thus these findings cannot be generalized to a more impaired group.

Of course, it is also possible that individuals who experienced decreases in physical function rehomed their pets, and this led to the apparent relationship of not owning a pet to deterioration in physical function. The data are less supportive of this idea because the association of pet ownership with the maintenance of physical function was present for both cat and dog owners and was not attributable to dog walking.

**Figure 3.**
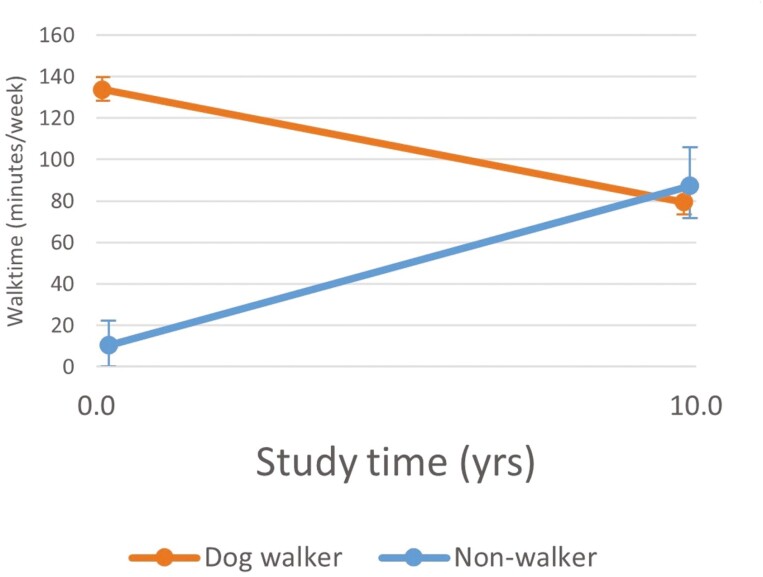
Changes in dog owners’ weekly time spent walking according to dog walking status with 10 years of aging, estimates based on linear mixed models that include age and comorbidities as covariates (*n* = 73).

Several previous cross-sectional studies associated dog ownership, and dog walking in particular, with more physical activity or better physical function (Christian et al., 2013; Coleman et al., 2008; Dall et al., 2017; Taniguchi et al., 2019; Thorpe et al., 2006). They differ from the current study that focuses on the trajectory of change, rather than the relative amount of, physical activity. In fact, the graphs suggest that there are differences in some measures of physical activity at specific time points. For example, Figure 3 demonstrates that at index visit, dog walkers spent more time walking than did dog owners who did not walk their dogs. However, this difference disappeared as the participants aged.

Use of linear mixed models with random intercepts enhances the interpretation of differences in changes in outcomes according to pet ownership-related characteristics. We treat pet ownership as a time-variant covariate. Participants only count as pet owners during the assessment occasions when they had a pet. This minimizes the impact of participants rehoming their pets on the results of the analysis. They are considered as nonowners in the next assessment. Furthermore, the random intercept models used for analysis, provide a unique baseline for each person which is used to examine trajectories of change independent of the starting point. This minimizes the effects of participants’ overall activity levels on changes in physical function.

### Limitations

Using the BLSA participants for this study had the advantage of a large well-described group of older adults with an extensive longitudinal objective evaluation of physical function. However, this sample is more affluent, White, and educated that the general aging population. The findings in this sample should be replicated in a more representative sample. This study examines dog relationships and dog related activities at one point in time, the survey visit. Data about dog walking may not accurately reflect the entire period over which physical function was assessed. Furthermore, attachment to the pet and the amount of time spent dog walking were not included in the analyses. These variables may have an impact on the magnitude of the physical benefits from pet ownership. The BLSA data are collected over time, allowing for longitudinal examination of change in physical function. Additional longitudinal assessment of attachment to pets and dog walking will permit future evaluation of their relationships with changes in physical function. Self-selection bias is a factor in this study because individuals are not randomly assigned to pet ownership groups. This limits causal inferences.

## Conclusion

This study provided longitudinal evidence suggesting that pet ownership moderates age-related declines in physical functional status later in life. Pet ownership was associated with less deterioration with aging over a mean of 7.5 years in five of the six measures of physical function, including the Health ABC Physical Function Battery, in community-dwelling older adults. There was no evidence that the association of dog and cat ownership with physical function differed or that dog walking was related to changes in physical function with aging. There also no evidence that pet ownership moderated age-related declines in leisure time physical activity in this population.

## Supplementary Material

igac080_suppl_Supplementary_Material
