# Supplementary material for: Pet Ownership and Maintenance of Physical Function in Older Adults—Evidence From the Baltimore Longitudinal Study of Aging (BLSA)
Source: Innov Aging. 2022 Dec 25;7(1):igac080. doi: 10.1093/geroni/igac080 (PMC10006577; doi:10.1093/geroni/igac080)
Supplement: igac080_suppl_Supplementary_Material [file igac080_suppl_supplementary_material.docx]

**Supplemental Table 1. Demographic and pet ownership characteristics of respondents at the time of the Baltimore Longitudinal Study of Aging** **index visit (follow up = 0 years)**

| **Characteristic** | **Overall (n=637)** | | | | **Non-pet owner (n=449)** | | | | **Pet owner (n=188)** | | | | **Test Statistic** | ***p*** |
| --- | --- | --- | --- | --- | --- | --- | --- | --- | --- | --- | --- | --- | --- | --- |
|  | **N** | **%** | **M** | **SD** | **N** | **%** | **M** | **SD** | **N** | **%** | **M** | **SD** |  |  |
| Dog owner | 84 | 13.19 |  |  | 0 | n/a |  |  | 84 | 44.68 |  |  |  |  |
| Cat owner | 67 | 10.52 |  |  | 0 | n/a |  |  | 67 | 35.64 |  |  |  |  |
| Age in years ^a^ |  |  | 68.25 | 9.64 |  |  | 69.47 | 9.67 |  |  | 65.33 | 8.97 | 5.03 | **<0.001** |
| Black ^b^ | 178 | 27.94 |  |  | 148 | 32.96 |  |  | 30 | 15.96 |  |  | 19.03 | **<0.001** |
| Female | 281 | 44.11 |  |  | 200 | 44.54 |  |  | 81 | 43.09 |  |  | 0.11 | 0.735 |
| < College Graduate ^b^ | 80 | 12.62 |  |  | 66 | 14.47 |  |  | 14 | 7.49 |  |  | 6.40 | **0.042** |
| Married or partnered ^b^ | 427 | 67.35 |  |  | 284 | 63.53 |  |  | 143 | 76.47 |  |  | 10.03 | **0.002** |
| Live Alone ^b^ | 163 | 25.79 |  |  | 128 | 28.70 |  |  | 35 | 18.82 |  |  | 6.70 | **0.009** |
| Live in single family house ^c^ | 525 | 82.94 |  |  | 348 | 77.85 |  |  | 177 | 95.16 |  |  |  | **<0.001** |
| Family income exceeds $50,000^b^ | 508 | 81.94 |  |  | 349 | 80.41 |  |  | 159 | 85.48 |  |  | 2.32 | 0.127 |
| Currently works ^b^ | 335 | 52.84 |  |  | 222 | 49.66 |  |  | 113 | 60.43 |  |  | 6.00 | **0.014** |
| Currently volunteers ^b^ | 372 | 58.68 |  |  | 263 | 58.84 |  |  | 109 | 58.29 |  |  | 0.001 | 0.991 |
| Body mass index ^a^ |  |  | 26.80 | 4.47 |  |  | 26.85 | 4.46 |  |  | 26.71 | 4.51 | 0.38 | 0.700 |
| Obese ^b^ | 133 | 20.88 |  |  | 98 | 21.83 |  |  | 35 | 18.62 |  |  | 0.83 | 0.363 |
| Comorbidities ^a^ |  |  | 0.95 | 1.17 |  |  | 0.99 | 1.15 |  |  | 0.85 | 1.22 |  | **0.030** |
| 400-M Walk time(wln) ^4^ |  |  | 256.85 | 43.96 |  |  | 259.59 | 40.82 |  |  | 250.69 | 49.90 |  | **<0.001** |
| Usual speed ^a^ |  |  | 1.20 | 0.22 |  |  | 1.19 | 0.22 |  |  | 1.24 | 0.21 | -2.88 | **0.004** |
| Rapid speed ^a^ |  |  | 1.84 | 0.35 |  |  | 1.82 | 0.34 |  |  | 1.90 | 0.35 | -2.81 | **0.005** |
| Physical performance ^a^ |  |  | 2.69 | 0.44 |  |  | 2.66 | 0.43 |  |  | 2.77 | 0.45 |  | **0.005** |
| Physical well-being(rln)* ^d^ |  |  | 53.66 | 5.70 |  |  | 53.70 | 5.45 |  |  | 53.54 | 6.28 |  | 0.520 |
| Leisure activity ^d^ |  |  | 5,233.90 | 4,390.71 |  |  | 4,905.29 | 4,305.27 |  |  | 6,015.79 | 4,503.62 | -3.38 | **<0.001** |
| Leisure walking(ln) ^a^ |  |  | 3.67 | 3.05 |  |  | 3.55 | 3.11 |  |  | 3.94 | 2.90 | -1.46 | 0.145 |
| Brisk exercise(ln) ^a^ |  |  | 0.03 | 4.59 |  |  | -0.02 | 4.62 |  |  | 0.16 | 4.53 | -0.45 | 0.655 |
| Weekly exercise(ln) ^a^ |  |  | 1.66 | 4.40 |  |  | 1.67 | 4.43 |  |  | 1.62 | 4.34 | 0.14 | 0.890 |
| Recommended exercise ^b^ | 176 | 27.85 |  |  | 129 | 28.99 |  |  | 47 | 25.13 |  |  | 0.97 | 0.324 |

*Note.* 1 Chi-Square test; 2 Fischer’s exact test; 3 Student’s t-test; 4 Wilcoxon rank sum test

400-M Walk time = seconds to walk 400 meters, Usual speed = usual gait speed (meters/second), Rapid speed = rapid gait speed (meters/second), Physical performance = physical performance battery score, Physical well-being = SF-12 physical function component score, Leisure activity = total leisure time physical activity (calories) per week, Leisure walking = minutes of leisure walking per week, Brisk exercise = minutes of “brisk” or exercise related walking per week, Weekly exercise= minutes spent exercising per week, Recommended exercise = dichotomous indicator for achieved recommended 150 minutes of moderate intensity exercise per week, (ln) = natural log transformed, (rln) = reflected and natural log transformed, (wln) = winsorized and natural log transformed.

* score reflected, lower is better.

^a^ Student’s t-test; ^b^ Chi-Square test; ^c^ Fischer’s exact test; ^d^ Wilcoxon rank sum test.

**Supplemental Table 2. Unconditional intraclass correlation coefficients (ICC) to assess dependence for physical function variables**

| **Outcome** | **ICC** |
| --- | --- |
| 400-M Walk time(wln) | 0.740525 |
| Physical performance | 0.752905 |
| Usual speed | 0.656342 |
| Rapid speed | 0.706012 |
| Physical well-being(rln) | 0.442900 |
| Leisure activity | 0.616180 |
| Leisure walking(ln) | 0.384170 |
| Brisk exercise(ln) | 0.388775 |
| Weekly exercise(ln) | 0.445852 |
| Recommended exercise | 0.420673 |

*Note.* 400-M Walk time = seconds to walk 400 meters, Usual speed = usual gait speed (meters/second), Rapid speed = rapid gait speed (meters/second), Physical performance = physical performance battery score, Physical well-being = SF-12 physical function component score, Leisure activity = total leisure time physical activity (calories) per week, Leisure walking = minutes of leisure walking per week, Brisk exercise = minutes of “brisk” or exercise related walking per week, Weekly exercise= minutes spent exercising per week, Recommended exercise = dichotomous indicator for achieved recommended 150 minutes of moderate intensity exercise per week, (ln) = natural log transformed, (rln) = reflected and natural log transformed, (wln) = winsorized and natural log transformed * score reflected, lower is better

**Supplemental Table 3. Outcome of bivariate linear mixed models showing changes in physical function as individuals aged (N=637)**

| **Outcome** | **Parameter estimate** | **se** | ***p*** |
| --- | --- | --- | --- |
| 400-M Walk time(wln) | 0.021 | 0.000 | **<0.001** |
| Usual speed | -0.011 | 0.001 | **<0.001** |
| Rapid speed | -0.027 | 0.000 | **<0.001** |
| Physical performance | -0.029 | 0.001 | **<0.001** |
| Physical well-being(rln)* | 0.013 | 0.001 | **<0.001** |
| Leisure activity | -188.166 | 10.914 | **<0.001** |
| Leisure walking(ln) | -0.063 | 0.013 | **<0.001** |
| Brisk exercise(ln) | -0.112 | 0.018 | **<0.001** |
| Weekly exercise(ln) | -0.100 | 0.017 | **<0.001** |
| Recommended exercise | -0.049 | 0.014 | **<0.001** |

*Note.* se = standard error, 400-M Walk time = seconds to walk 400 meters, Usual speed = usual gait speed (meters/second), Rapid speed = rapid gait speed (meters/second), Physical performance = physical performance battery score, Physical well-being = SF-12 physical function component score, Leisure activity = total leisure time physical activity (calories) per week, Leisure walking = minutes of leisure walking per week, Brisk exercise = minutes of “brisk” or exercise related walking per week, Weekly exercise= minutes spent exercising per week, Recommended exercise = dichotomous indicator for achieved recommended 150 minutes of moderate intensity exercise per week, (ln) = natural log transformed, (rln) = reflected and natural log transformed, (wln) = winsorized and natural log transformed.

*score reflected, lower is better.

**Supplemental Table 4. Estimates for interaction parameters from linear mixed models examining the independent associations of dog ownership and cat ownership to changes in physical function variables with aging, adjusted for age and comorbidity (n=146)**

| **Outcome** | **Interaction** | **with** | **Follow-up** |  | **Interaction** | **With** | **Follow-up** |
| --- | --- | --- | --- | --- | --- | --- | --- |
|  | **DO est** | **DO se** | **DO *p*** |  | **CO est** | **CO se** | **CO *p*** |
| 400-M Walk time(wln) | -0.002 | 0.001 | 0.135 |  | -0.005 | 0.001 | **<0.001** |
| Physical performance | 0.009 | 0.004 | **0.044** |  | 0.015 | 0.004 | **<0.001** |
| Usual speed | 0.002 | 0.002 | 0.257 |  | 0.002 | 0.002 | 0.322 |
| Rapid speed | 0.008 | 0.003 | **0.016** |  | 0.002 | 0.003 | 0.568 |
| Physical well-being(rln)* | -0.002 | 0.004 | 0.538 |  | -0.009 | 0.003 | **0.008** |
| Leisure activity | -33.861 | 36.852 | 0.358 |  | -6.771 | 35.229 | 0.848 |
| Leisure walking(ln) | 0.057 | 0.044 | 0.197 |  | -0.018 | 0.042 | 0.679 |
| Brisk exercise(ln) | -0.056 | 0.059 | 0.339 |  | 0.048 | 0.057 | 0.394 |
| Weekly exercise(ln) | -0.041 | 0.056 | 0.467 |  | 0.093 | 0.054 | 0.087 |
| Recommended exercise | 0.041 | 0.044 | 0.356 |  | -0.039 | 0.047 | 0.410 |

*Note.* DO = dog ownership, non-ownership is reference category, CO = cat ownership, non-ownership is reference category, est = parameter estimate, se = standard error, 400-M Walk time = seconds to walk 400 meters, Usual speed = usual gait speed (meters/second), Rapid speed = rapid gait speed (meters/second), Physical performance = physical performance battery score, Physical well-being = SF-12 physical function component score, Leisure activity = total leisure time physical activity (calories) per week, Leisure walking = minutes of leisure walking per week, Brisk exercise = minutes of “brisk” or exercise related walking per week, Weekly exercise= minutes spent exercising per week, Recommended exercise = dichotomous indicator for achieved recommended 150 minutes of moderate intensity exercise per week, (ln) = natural log transformed, (rln) = reflected and natural log transformed, (wln) = winsorized and natural log transformed.

* score reflected, lower is better

**Supplemental Table 5. Estimates for interaction parameters from linear mixed models examining the associations of index visit pet ownership, dog ownership, and cat ownership to changes in physical function variables with aging, adjusted for age and comorbidity (n=637)**

| **Outcome** | **Interaction** | **with** | **Follow-up** | **Interaction** | **With** | **Follow-up** | **Interaction** | **with** | **Follow-up** |
| --- | --- | --- | --- | --- | --- | --- | --- | --- | --- |
|  | **PO est** | **PO se** | **PO *p*** | **DO est** | **Do se** | **DO *p*** | **CO est** | **CO se** | **CO *p*** |
| 400-M Walk time(wln) | -0.004 | 0.001 | **<0.001** | 0.001 | 0.001 | 0.459 | -0.005 | 0.001 | **<0.001** |
| Physical performance | 0.137 | 0.003 | **<0.001** | 0.003 | 0.004 | 0.559 | 0.018 | 0.004 | **<0.001** |
| Usual speed | 0.003 | 0.001 | **0.017** | 0.004 | 0.002 | 0.060 | 0.003 | 0.002 | 0.122 |
| Rapid speed | 0.003 | 0.002 | 0.107 | 0.006 | 0.003 | **0.047** | 0.003 | 0.003 | 0.268 |
| Physical well-being(rln)* | -0.006 | 0.002 | **0.024** | -0.001 | 0.004 | 0.733 | -0.011 | 0.004 | **0.002** |
| Leisure activity | -37.071 | 24.709 | 0.134 | -37.255 | 36.562 | 0.308 | -14.249 | 37.063 | 0.701 |
| Leisure walking(ln) | 0.028 | 0.030 | 0.350 | 0.064 | 0.044 | 0.144 | -0.020 | 0.045 | 0.649 |
| Brisk exercise(ln) | 0.003 | 0.040 | 0.946 | -0.065 | 0.059 | 0.265 | 0.071 | 0.060 | 0.242 |
| Weekly exercise(ln) | 0.009 | 0.038 | 0.822 | -0.024 | 0.056 | 0.667 | 0.079 | 0.058 | 0.171 |
| Recommended exercise | 0.030 | 0.031 | 0.337 | 0.112 | 0.043 | **0.009** | -0.055 | 0.051 | 0.280 |

*Note.* PO =pet ownership, non-ownership is reference category, DO = dog ownership, non-ownership is reference category, CO = cat ownership, non-ownership is reference category, est = parameter estimate, se = standard error, 400-M Walk time = seconds to walk 400 meters, Usual speed = usual gait speed (meters/second), Rapid speed = rapid gait speed (meters/second), Physical performance = physical performance battery score, Physical well-being = SF-12 physical function component score, Leisure activity = total leisure time physical activity (calories) per week, Leisure walking = minutes of leisure walking per week, Brisk exercise = minutes of “brisk” or exercise related walking per week, Weekly exercise= minutes spent exercising per week, Recommended exercise = dichotomous indicator for achieved recommended 150 minutes of moderate intensity exercise per week, (ln) = natural log transformed, (rln) = reflected and natural log transformed, (wln) = winsorized and natural log transformed.

* score reflected, lower is better.
